# Optimized expression and enhanced production of alkaline protease by genetically modified *Bacillus licheniformis* 2709

**DOI:** 10.1186/s12934-020-01307-2

**Published:** 2020-02-24

**Authors:** Cuixia Zhou, Huiying Zhou, Dengke Li, Huitu Zhang, Hongbin Wang, Fuping Lu

**Affiliations:** Key Laboratory of Industrial Fermentation Microbiology, Ministry of Education, College of Biotechnology, Tianjin University of Science & Technology, No. 29, 13th Road, Tianjin Economic-Technological Development Area, Tianjin 022, 300457 People’s Republic of China

**Keywords:** *Bacillus licheniformis*, Alkaline protease, Markerless gene editing, Host modification, Gene expression

## Abstract

**Background:**

*Bacillus licheniformis* 2709 is extensively applied as a host for the high-level production of heterologous proteins, but *Bacillus* cells often possess unfavorable wild-type properties, such as production of viscous materials and foam during fermentation, which seriously influenced the application in industrial fermentation. How to develop it from a soil bacterium to a super-secreting cell factory harboring less undomesticated properties always plays vital role in industrial production. Besides, the optimal expression pattern of the inducible enzymes like alkaline protease has not been optimized by comparing the transcriptional efficiency of different plasmids and genomic integration sites in *B. licheniformis*.

**Result:**

*Bacillus licheniformis* 2709 was genetically modified by disrupting the native *lchAC* genes related to foaming and the *eps* cluster encoding the extracellular mucopolysaccharide via a markerless genome-editing method. We further optimized the expression of the alkaline protease gene (*aprE*) by screening the most efficient expression system among different modular plasmids and genomic loci. The results indicated that genomic expression of *aprE* was superior to plasmid expression and finally the transcriptional level of *apr*E greatly increased 1.67-fold through host optimization and chromosomal integration in the vicinity of the origin of replication, while the enzyme activity significantly improved 62.19% compared with the wild-type alkaline protease-producing strain *B. licheniformis*.

**Conclusion:**

We successfully engineered an AprE high-yielding strain free of undesirable properties and its fermentation traits could be applied to bulk-production by host genetic modification and expression optimization. In summary, host optimization is an enabling technology for improving enzyme production by eliminating the harmful traits of the host and optimizing expression patterns. We believe that these strategies can be applied to improve heterologous protein expression in other *Bacillus* species.
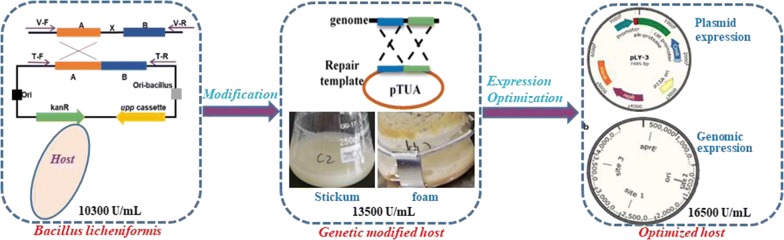

## Background

Alkaline protease has been widely used in industry and everyday products, which resulted in continuously increasing need for cost-effective production of this enzyme [1]. *Bacillus* species are the major industrial protease producers, among which *Bacillus licheniformis* 2709 has been proven to be a promising alkaline protease producer due to its easy cultivation, GRAS status and strong ability to secrete proteins directly into the extracellular medium [2]. However, as a microorganism from the upper layers of the soil or plant rhizosphere, *B. licheniformis* has many undesirable wild-type properties, such as sporulation under nutrient depletion conditions, as well as the production of large amounts of foam and viscous substances to increase the cellular competitiveness and survival in the challenging environment [3–5]. These intrinsic features lead to significant contamination risks and high production costs in industrial fermentations. Many studies were conducted to solve these problems and thereby decreased the requirements and difficulties in industrial operations. A common and effective method involves the deletion of undesirable intrinsic genes, including *spoIIAC* (related to spore formation) [2, 6] and *srfAC* (related to foaming) [7, 8], which led to a significant improvement of the traits in the engineered strains. Consequently, it is greatly necessary and valuable to construct an advanced chassis based on *B. licheniformis* cells for industrial applications, without the undesirable wild-type properties.

In addition to these host-modification strategies to improve the cellular performance, some gene regulation and expression methods have also been applied to increase protein production. In recent years, a great amount of fundamental work on promoters has been undertaken, and various promoters have been identified and reconstructed to achieve high-level expression of recombinant proteins, with some notable results [9–11]. Although transcription is the first and key step in the process of gene expression [12], the transcriptional effectiveness of one promoter varies for different proteins, and the so-called optimal promoter sequences cannot be generalized for all heterologous enzymes [13, 14]. Consequently, some researchers have considered the compatibility between expression elements and the host for the production of enzymes, and have intensively investigated the endogenous expression elements, combining suitable expression patterns to reduce host-intrinsic expression bottlenecks and thereby improve protein production [15–17]. It is well known that effective gene expression patterns play a pivotal role in the progression from the lab bench toward industrial applications. A series of plasmid systems were developed in *Bacillus*, mostly relying on multicopy replication origins to produce massive amounts of recombinant proteins [18]. However, not all genes are suitable for expression from high-copy-number plasmids, and some studies fail to obtain enhanced expression of the target gene, in addition to the well-known problems of plasmid stability and antibiotics-based selection [19]. However, if the target protein has a more effective expression level in line with the native genomic genes, expression via genomic integration can ensure the stability of genetic material [20]. Previous studies found that differences in chromosomal gene expression levels are highly correlated with the position relative to the replication origin [18, 21]. To our best knowledge, the expression level of *aprE* has not been optimized by comparing the transcriptional efficiency of different plasmids and genomic integration sites in *B. licheniformis.*

In the study, we applied a genome editing method with counter-selectable markers based on a temperature-sensitive plasmid to engineer the host by genetically eliminating undesired wild-type features and optimizing the expression patterns. The expression levels of the endogenous expression cassette *aprE* in different plasmids and genomic loci was further investigated.

## Materials and methods

### Strains and culture conditions

All the strains and plasmids used in this study are listed in Table 1. The *B. licheniformis* strain BL Δupp was used as the starting strain for genetic modifications; *E. coli* strain EC135 was employed as the donor strain for plasmid construction and the *E. coli* strain EC135 pM.Bam was used for DNA methylation [22]. The shuttle vectors pWH1520 and pLY-3 were used to construct *aprE* expression vectors. The temperature-sensitive shuttle vector pKSVT was used to construct the gene knockout vectors.

**Table 1:** Strains and plasmids used in the study

Strain or plasmid	Characteristics or purpose	Reference
Strains		
*E. coli* EC135	Knockout vectors construction	Chinese Academy of Science
*E. coli* EC135 pM.Bam	Plasmid DNA methylation modification	Chinese Academy of Science
*B. licheniformis* 2709	Wild strain	CICC
*B. licheniformis* 2709 Δupp (BL Δupp)	Parent host	[23]
*B. licheniformis* Δapr (BL ΔA)	Δ*aprE*, *aprE* gene deletion	This work
*B. licheniformis* Δeps (BL ΔEP)	Δ*eps*, *eps cluster* deletion	This work
*B. licheniformis* Δlch (BL ΔS)	Δ*lchAC*, *lchAC* gene deletion	This work
*B. licheniformis* Capr (BL CA)	*aprE* gene complementation	This work
*B. licheniformis* Clch (BL CS)	*lchAC* gene complementation	This work
*B. licheniformis* ΔepsΔlch (BL ΔES)	Δ*eps,* Δ*lchAC*	This work
*B. licheniformis* ΔepsΔlchΔapr (BL ΔESA)	Δ*eps,* Δ*lchAC,* Δ*aprE*	This work
BL ΔESA-pWHA	Recombinant strain with pWHA of BL ΔESA	This work
BL ΔESA-pWH1520	BL ΔESA harboring pWH1520, control	This work
BL ΔESA-pLYA	Recombinant strain with pLYA of BL ΔESA	This work
BL ΔESA-pLY-3	BL ΔESA harboring pLY-3, control	This work
*B. licheniformis I1* (BL I1)	Integration expression of *aprE* in site1 in BL ΔESA	This work
*B. licheniformis I2* (BL 12)	Integration expression of *aprE* in site2 in BL ΔESA	This work
*B. licheniformis I3* (BL I3)	Integration expression of *aprE* in site3 in BL ΔESA	This work
Plasmids		
pWH1520	Shuttle expression vector, Amp^r^ (*E. coli*) and Tet^r^ (*Bacillus*): MCS	Nankai University
pLY-3	Shuttle expression vector, Kana^r^ (*E. coli*) and Cm^r^ (*Bacillus*): MCS	Lab collection
pKSVT	Temperature-sensitive shuttle plasmid, Kana^r^	Hubei University
pTU	pKSVT, *upp* gene	This work
pTUA	Knockout vector, *aprE* gene deletion	This work
pTUE	Knockout vector, *eps cluster* deletion	This work
pTUS	Knockout vector, *lchAC* gene deletion	This work
pTUCA	Backcrossed vector, *aprE* gene complementation	This work
pTUCS	Backcrossed vector, *lchAC* gene complementation	This work
pTUAI 1	pTU, integrating *aprE* expression cassette into site 1	This work
pTUAI 2	pTU, integrating *aprE* expression cassette into site 2	This work
pTUAI 3	pTU, integrating *aprE* expression cassette into site 3	This work
pWHA	pWH1520, *aprE* expression cassette	This work
pLYA	pLY-3, *aprE* expression cassette	This work

Luria–Bertani (LB) medium was used for the cultivation of *Bacillus* and *E. coli*, with antibiotics (100 mg/L ampicillin, 50 mg/L spectinomycin, 20 mg/L tetracycline, 30 mg/L 5-fluorouracil, 50 mg/L kanamycin) where appropriate. The *E. coli* and *Bacillus* strains were all grown at 37 °C with aeration, except for the plasmid integration/excision experiments, which were conducted at 45 °C. For the production of alkaline protease, the seed culture was grown in 50 mL LB medium at 37 °C until the OD_600_ reached ~ 1.0, and then transferred into 100 mL of fermentation medium at a 2% inoculation rate. The AprE fermentation medium contained corn starch (64 g/L), soybean meal (40 g/L), Na_2_HPO_4_ (4 g/L), KH_2_PO_4_ (0.3 g/L), and thermostable amylase (0.7 g/L) (Biotopped, Beijing, China), pH 7.2.

### Plasmid construction

The plasmids and primers used in this study are listed in Table 1 and Additional file 1: Table S1, respectively. An 845-bp DNA fragment carrying the *upp* gene with its promoter was generated by PCR amplification from a previously constructed vector of the CRISPR system using the primer pair PU-F/PU-R. After digesting with *Kpn*I and *Sal*I, the fragment was cloned in the *Kpn*I/*Sal*I sites of pKSVT, resulting in a counter-selectable plasmid designated pTU.

As an example, the *aprE* deletion strain was constructed as follows: For construction of the deletion plasmids, the up- and downstream homologous arms (~ 500 bp, LH and RH) for *aprE* gene deletion were obtained using the primer pairs Apr-LF/Apr-LR and Apr-RF/Apr-RR, respectively, and were cloned between the *Bam*HI/*Sac*II sites of pTU by fusion cloning to generate the knockout vector pTUA. The construction of other knockout vectors was accomplished in the same way. The integrative *aprE* expression vector pTUAI was constructed in analogy to the knockout vector. The *aprE* expression cassette harboring the *aprE* gene with its own 5′ regulatory region and 3′ transcription terminator was inserted between the up- and downstream homologous arms to be integrated into the different chromosomal target sites of the *aprE* deficient strain by recombination. Complementary plasmids were constructed in the same way to obtain the backcrossed strains.

For investigating the plasmid-mediated expression levels, the *aprE* expression cassette amplified by PCR using primers AP-F1/AP-R1 from the genome of *B. licheniformis* was individually cloned between the *Sac*I/*Kpn*I sites of pWH1520 with low copy number and the *Kpn*I/*Bgl*II sites of pLY-3 with high copy number (AP-F2/AP-R2) by fusion cloning to form pWHA and pLYA, respectively.

### Gene knockout and genetic complementation in *B. licheniformis*

To construct the alkaline protease deficient host by disrupting the *aprE* gene encoding alkaline protease, the deletion plasmid pTUA was methylated and transferred into BL Δupp by electroporation. The Kan^R^ positive transformants were picked and cultivated in glass tubes containing 5 mL LB with kanamycin for about 10 h at 45 °C to facilitate plasmid integration. Then, the culture was spread on LB agar plates with kanamycin and incubated for about 10 h at 45 °C. The primers Apr-VF/T-R was used to screen single-crossover recombinants by colony PCR. The correct band size was approximately the size of LH + RH when integrated at LH in the genome or the size of LH + apr + RH when integrated at RH in the genome. The successful single-crossover recombinant was selected and cultivated in a tube with 5 mL LB for about 12 h at 37 °C (usually with two transfers, 10 µL culture to next tube) to facilitate the second recombinational event and plasmid excision. The culture was then spread on LB agar plates containing 5-FU and incubated at 37 °C for about 16 h. Diagnostic PCR reactions were performed using the primers Apr-VF/Apr-VR designed according to the genomic sequences flanking the homologous arms and were further verified by DNA sequencing.

The other genes were deleted, replaced or integrated into the genome analogously to the example. Similarly, the backcrossed strains were individually constructed using the specific backcross vectors into the mutants using the same gene editing method.

### Construction of the recombinant strains

The verified recombinant expression plasmid pWHA (tet^R^) or pLYA (kan^R^) carrying the *aprE* expression cassette including its encoding gene of 1140 bp and upstream regulatory region of ~ 400 bp (5′ to 3′) was electroporated into the *aprE* deficient host BL ΔA, a mutant obtained in the study. Tetracycline- or kanamycin-resistant transformants were selected and confirmed by colony PCR. And the positive colonies were picked to investigate the expression level (enzyme activity and transcriptional level) of the *aprE* gene.

The integrative expression plasmid carrying the *aprE* expression cassette was used to construct the recombinant strain with integrated *aprE* using the gene editing approach. Three specific loci of the genome were selected according to our transcriptome analysis at different enzyme synthesis stages performed by us and others in previous studies [18, 21]. The first genomic location was near the origin of replication (Ori); the second locus is the symmetrical position of the *aprE*; the third target site is the symmetrical position of the Ori. Finally, we observed the alkaline protease enzyme activity and transcriptional levels of the different confirmed mutants.

### Analytic methods

In order to inspect the cell growth of the strains, an independent colony was picked into 50 mL of fresh liquid LB medium and cultivated at 37 °C and 220 rpm. The optical density at 600 nm (OD_600_) of the bacterial suspension (200 μL/microtiter well) was measured using the Infinite 200PRO Laboratories microplate reader (TECAN, Austria). The quantification of viable bacteria was performed to investigate the biomass accumulation [2], and changes of the control strain and the mutant losing capacity of forming viscous materials during the fermentation process. Three measurements were conducted for each sample.

The production of alkaline protease of the different strains in the study was determined using samples at different cultivation times in shake-flask fermentations. Because the alkaline protease activity had obviously positive correlation with the *aprE* expression quantity, the alkaline protease activity in culture supernatants was investigated using the detection method published by the national standardization administration commission [23].

The viscous substance was presumed to be exopolysaccharides (EPS) or polyglutamic acid (PGA) according to previous research [24–27], and it was identified via gas chromatography-mass spectrometry (GC/MS) (Agilent, USA). Before GC/MS detection, the samples were treated as follows: The supernatant of the fermentation broth was successively collected, diluted, monomeric sugars removed by ultrafiltration, and the interception was combined with a triple volume of 70% ethanol for 30 min. After centrifugation, the supernatant was naturally dried in the fume hood, and then 2 mL of 2 M trifluoroacetic acid was added and then transferred into an ampoule bottle and sealed, followed by acidolysis for 3 h at 120 °C. Finally, the reaction liquid was dried using a SBHCONC/1 pressure blowing concentrator (Stuart, England). The parameters of GC/MS were set as follows: The injector and detector temperatures were kept at 260 °C. A sample comprising 1 μL sample was injected into an HP-INNOWAX column (30 m × 0.250 mm i.d., 0.25 μm film thickness, Agilent). Helium was used as the carrier gas at a flow rate of 1 mL/min. The GC oven temperature was initially held at 60 °C for 2 min, then raised to 290 °C with a temperature ramp of 5 °C/min, and then raised to 310 °C at a rate of 10 °C/min and kept for 8 min.

### Analysis of transcription levels

The strains were cultured in fermentation medium for 48 h at 37 °C and the cells were collected at the stable phase of alkaline protease activity. Total RNA was extracted using TRIzol^®^ Reagent (Promega, USA). The quality of the RNA was assessed by agarose gel electrophoresis and the total RNA concentration was determined using a NanoDrop 1000 spectrophotometer (Thermo Scientific, USA). RNase-free DNase I (TaKaRa, Japan) was used to digest trace DNA, and the first strand of cDNA was synthesized using RevertAid First Strand cDNA Synthesis Kit (Thermo, USA). The quantitative real-time PCR (qRT-PCR) were performed using SYBR^®^ Premix Ex Taq™ II (TaKaRa, Japan) in an ABI Stepone Real-Time PCR System (Stepone plus, Thermo Scientific, USA). The primers listed in the Additional file 1: Table S1 were used to amplify the alkaline protease gene (AP-F/AP-R) from the parent strain and the other mutants. The 16S rRNA (S-F/S-R) of *B. licheniformis* was used as the internal reference to normalize the data. The transcriptional levels of the alkaline protease gene in different recombinant strains and the control strain BL *Δupp* were investigated and compared using the 2^−ΔΔCt^ method. All the experiments were performed in triplicate.

### Statistical analyses

All experiments were performed in triplicate, and the experimental data were expressed as the means ± standard deviations. The significance of differences was evaluated using two-way ANOVA with P < 0.05.

### Nucleotide sequence accession number

The sequence of the *apr*E expression cassette and the relevant homologous repair sequence has been deposited in GenBank under the Accession Number CP033218.

## Results

### Identification of the viscous substance produced by *B. licheniformis* using GC/MS

**Fig. 1:**
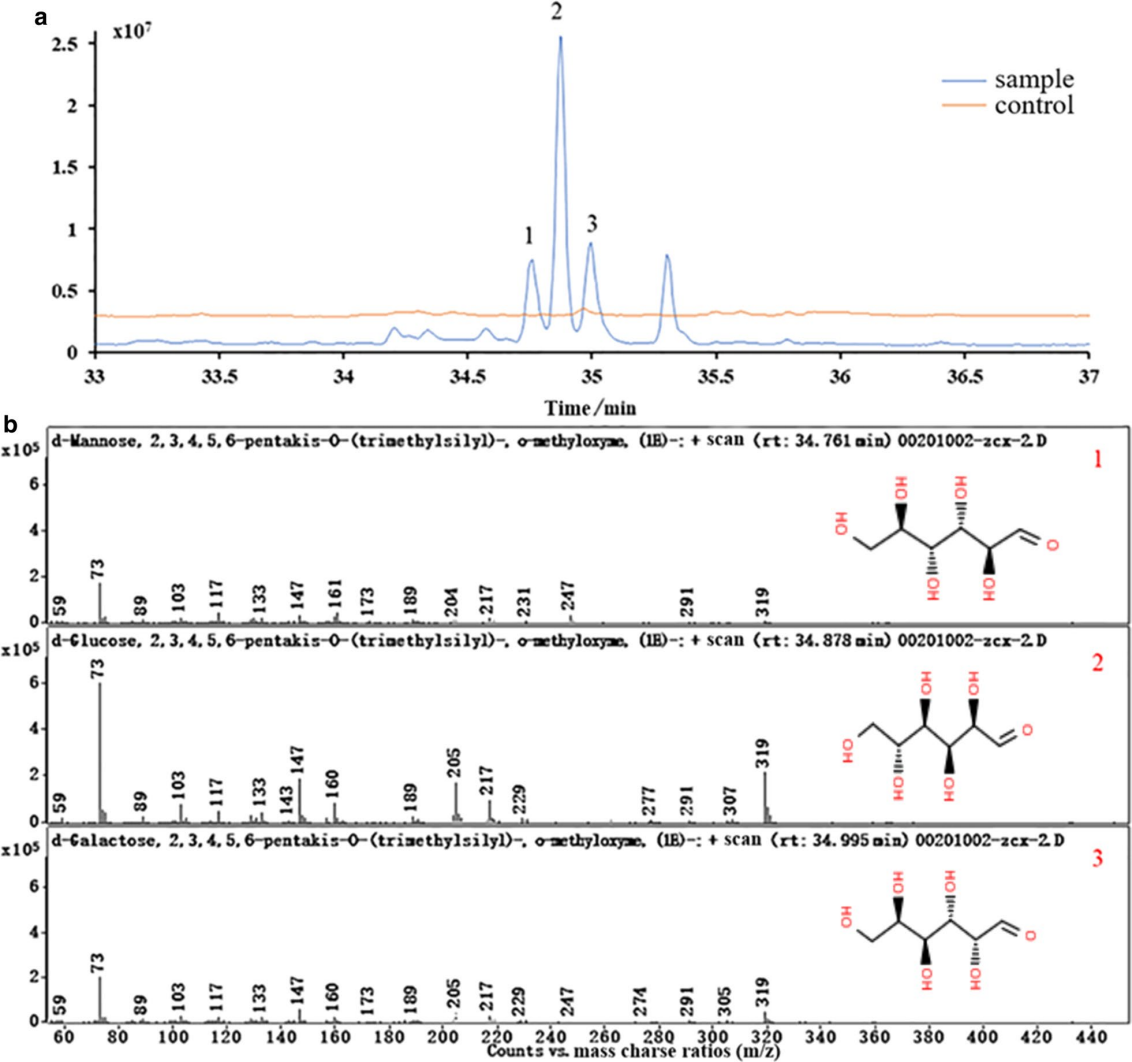
Total ion chromatogram and mass spectrum of the major monosaccharides detected in *B. licheniformis*. **a** Sample, represented the processed product of the EPS-producing strain (BL Δupp) to be detected by GC/MS; control, represented processed product of the fermentation medium to be detected by GC/MS; **b**-1 the mannose was identified by mass spectrum with a matching quality of 94.68% comparing with the NIST-17 Database; **b**-2 the glucose was identified by mass spectrum with a matching-degree of 90.36% comparing with the Database; **b**-3 the galactose was identified by mass spectrum with a matching-degree of 91.07% comparing with the database

**Fig. 2:**
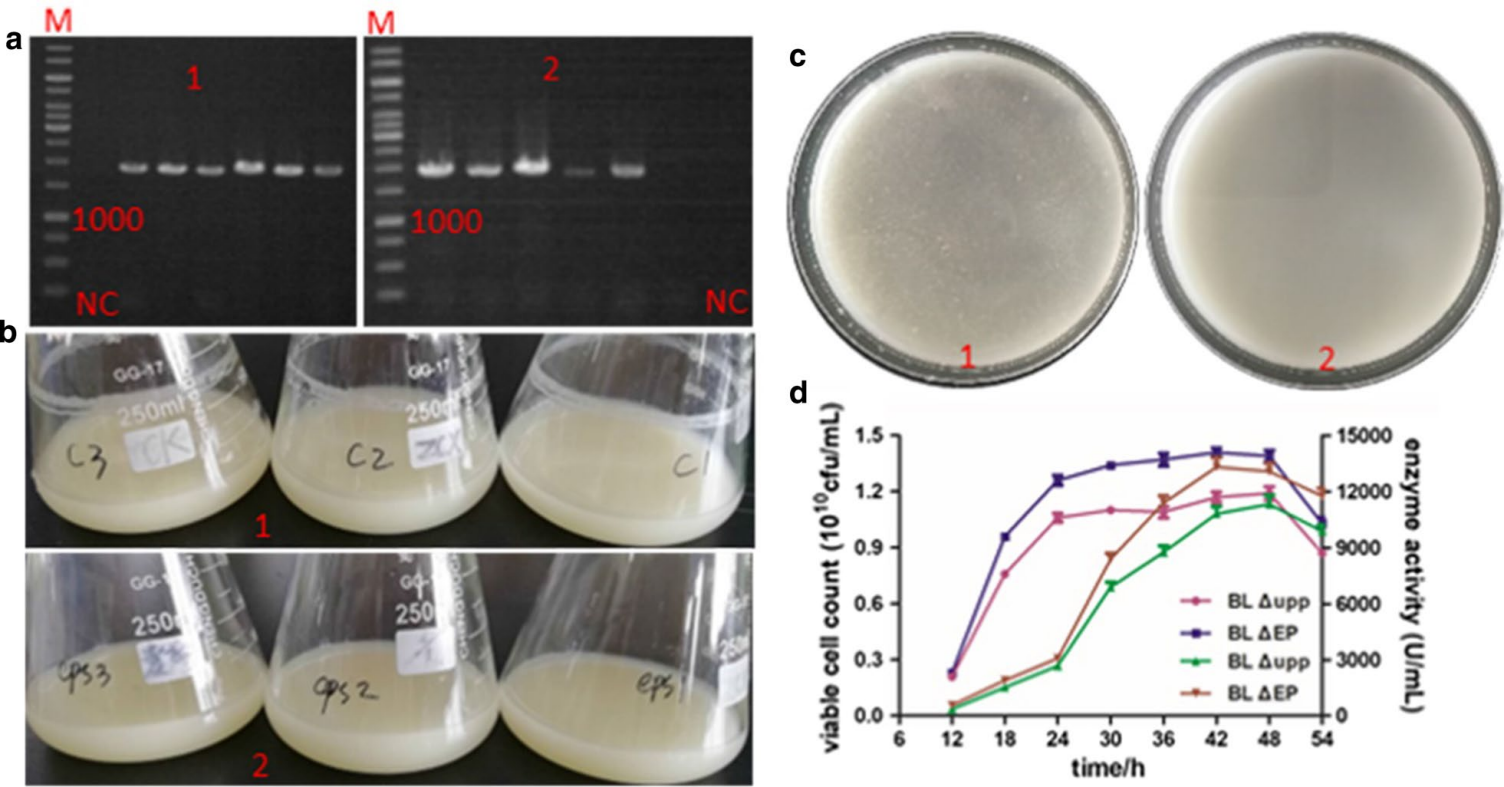
Confirmation of the *eps* cluster disruption and difference comparison of phenotype**. a** Screening process of the mutants. **a**-1 was the verification of the single-crossover recombinant with a band of 1750 bp and **a**-2 was the verification of the double-crossover mutant with a band of 1900 bp. M-marker, 250, 500, 750, 1000, 1500, 2000, 2500, 3000, 4000, 5000, 6000, 8000, 10,000 bp; NC-negative control; **b** Comparation of cell growth in 250 mL flask with LB medium of different strains. **b**-1 was the thallus of the wild-strain and **b**-2 was the thallus of the *eps* cluster mutant; **c** Fermentation broth of the *eps* cluster mutant and the wild-type strain. **c**-1 was the fermentation broth with granulated thallus of wild-type strain and **c**-2 was the exquisite fermentation broth of *eps* cluster mutant; **d** Alkaline protease enzyme activity assay and the viable cell count of the *eps* cluster mutant and wild-type strain. The left Y axis indicated the viable cell count (  BL Δupp,  BL ΔEP) and the right Y axis indicated the alkaline protease enzyme activity (  BL Δupp,  BL ΔEP)

**Fig. 3:**
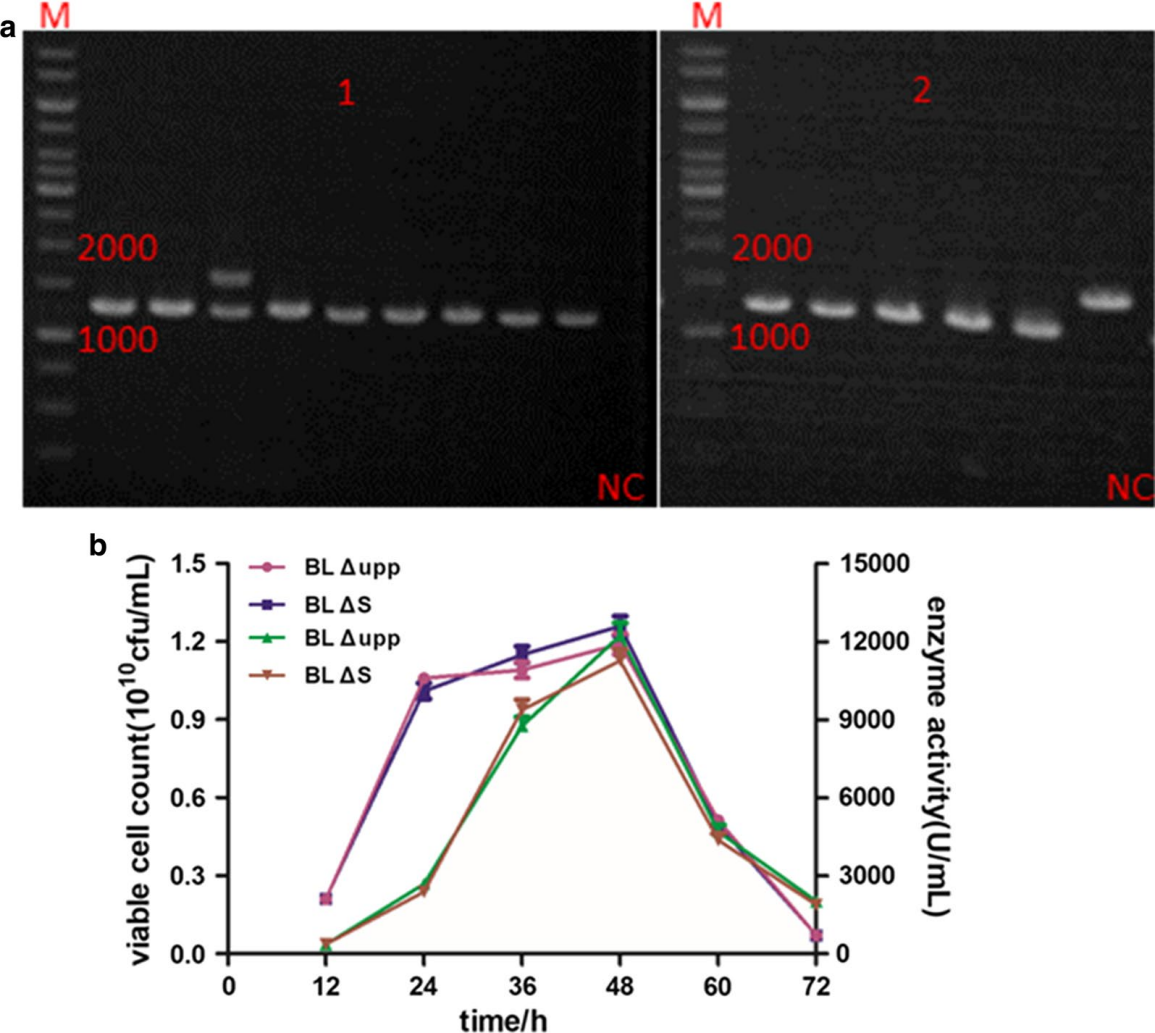
Verification of the *lchAC* disruption and the characterization of the mutant. **a** Screening process of the mutants. **a**-1 was the verification of the single-crossover recombinant with a band of 1200 bp and **a**-2 was the verification of the double-crossover mutant with a band of 1300 bp (*M* marker, *NC* negative control); **b** Alkaline protease enzyme activity assay and the viable cell count of the *lchAC* mutant and wild-type strain. The left Y axis indicated the viable cell count (  BL Δupp,  BL ΔS) and the right Y axis indicated the alkaline protease enzyme activity (  BL Δupp,  BL ΔS)

The sticky substance was tentatively considered to be either EPS or PGA according to previous studies [28]. In order to investigate the extracellular viscous substance produced in the fermentation medium of *B. licheniformis*, fermentation supernatant was collected after 48 h of cultivation and pretreated for detection. The samples were processed by alcohol precipitation and acidolysis, and the hydrolysis products were identified by GC/MS to analyze their retention times and mass fragmentation patterns (Fig. 1). As shown in the chromatogram and mass spectrum, three characteristic peaks of monosaccharides were isolated and identified by comparative a nalyzing the molecular weight and charge-mass ratio with the NIST-17 database. The characteristic peak of mannose (Fig. 1b-1) was matched in the database with a match quality of 94.68%; meanwhile, two distinct peaks of glucose (Fig. 1b-2) and galactose (Fig. 1b-3) was individually matched in the database with values of 90.36% and 91.07%. The matching-degree of three monosaccharides in mass spectrum were relatively high reaching the credibility when compared with the NIST-17 database. They are known as the key components of microbial extracellular heteropolysaccharides [9]. Furthermore, various amino acids were also detected but we found that glutamate was not prominent, which demonstrated that there was little or no PGA in the extracellular products, and further confirmed that the viscous substance was EPS but not PGA. Thus, it seemed possible to achieve an improvement of the host performance by elimination the formation of EPS.

### Genetic modification of the host

#### Disruption of *eps* gene cluster encoding EPS

The synthesis process of bacterial extracellular polysaccharides (EPS) is very complex because EPS synthesis is an integrated result of the cooperative actions of a great many gene products (Barcelos et al. 2019). To avoid the formation of mucus in the host, the *eps* cluster responsible for the synthesis of extracellular mucopolysaccharide in *B. licheniformis* was considered for deletion because it was identified as a nonessential region in a previous study [3]. The knockout vector pTUE was transferred into the BL Δupp strain, and the positive mutants were screened out using the genome editing procudure (Additional file 1: Fig. S1). An approximately 15-kbp fragment in the *eps* cluster was targeted to be removed from the chromosome in the strain using a HA of 1725 bp. As presented in Fig. 2a, the single-crossover recombinants were identified by colony PCR using the primer pair Eps-VF/T-R with a product size of 1785 bp (Fig. 2a-1), and the double-crossover mutants were verified by a PCR product of approximately 1900 bp (Fig. 2a-2) using the primer pair Eps-VF/Eps-VR. The mutants were further validated by DNA sequencing, and the corresponding phenotype of EPS formation, as indicated in Fig. 2b. The EPS synthesis ability of the generated mutant BL ΔEP as measured by GC/MS was significantly reduced (not detected), and there was no thallus on the flask wall as shown in Fig. 2b-2 compared with the parent strain BL Δupp (Fig. 2b-1) when cultivated in LB medium at 37 °C. An agglomeration-free and exquisite fermentation broth of BL ΔEP was obtained, as demonstrated in Fig. 2c.

The cell growth and production of alkaline protease were also investigated to evaluate the mutant’s cellular performance. As shown in Fig. 2d, the biomass accumulation (viable count) of BL ΔEP was slightly enhanced, probably due to an increase of the dissolved oxygen concentration, which spontaneously strengthened the production of alkaline protease (enzyme activity improved 25.32% compared with the control strain, BL Δupp). Furthermore, the maximum of enzyme activity in the BL ΔEP strain (at 42 h, 13,309 U/mL) was obtained 6 h earlier than that of the BL Δupp strain (at 48 h, 10,620 U/mL).

### Disruption of *lchAC* regulating surfactin synthesis

In addition to iturin and fengycin, surfactin has amphiphilic properties as a component of lipogenic peptide and its accumulation promotes foam production in *B. subtillis*. But lichenysin is mainly produced by *B. licheniformis*, which even though has one amino acid difference with surfactin, it has similar function with surfactin. Because the *srfAC* genes are crucial regulators of surfactin synthesis [7], the disrupting vector was designed to target this similar operon (*lchAC*) in *B. licheniformis*. The resulting *lchAC* mutant BL ΔS was obtained applying the same gene editing procedure as deleting the *eps* cluster. The mutant BL ΔS was screened by amplifying the up/and downstream regions (Lch-VF/Lch-VR) with the correct band of 1180 bp based on the successful single-crossover recombinant verified by diagnostic PCR using the primers Lch -VF/T-R (Fig. 3a) with a band of 1200 bp. To test the foam production of BL Δupp and BL ΔS, cells were cultivated in a 5-L fermenter with 3 L of fermentation medium for 72 h, and the cell growth showed no significant differences (Fig. 3b). The foam began to appear at 3 h, and reached a maximum at about 12 h, during which BL Δupp produced a large amount of foam and required the continuous addition of about 250 µL antifoam. By contrast, BL ΔS produced much less foam and needed only 30 µL of antifoam. Interestingly, the foam height of BL Δupp and BL ΔS was similar, but the foam of BL ΔS was more sensitive to antifoam. Additionally, the production of alkaline protease was not influenced by the elimination of *lchAC*. The backcrossed strain was constructed to verify the function of LchAC by introducing each of the specific backcrossed vectors including the complementary gene *lchAC* into the mutants using the same gene editing method.

### Establishment of *aprE* deficient strain

**Fig. 4:**
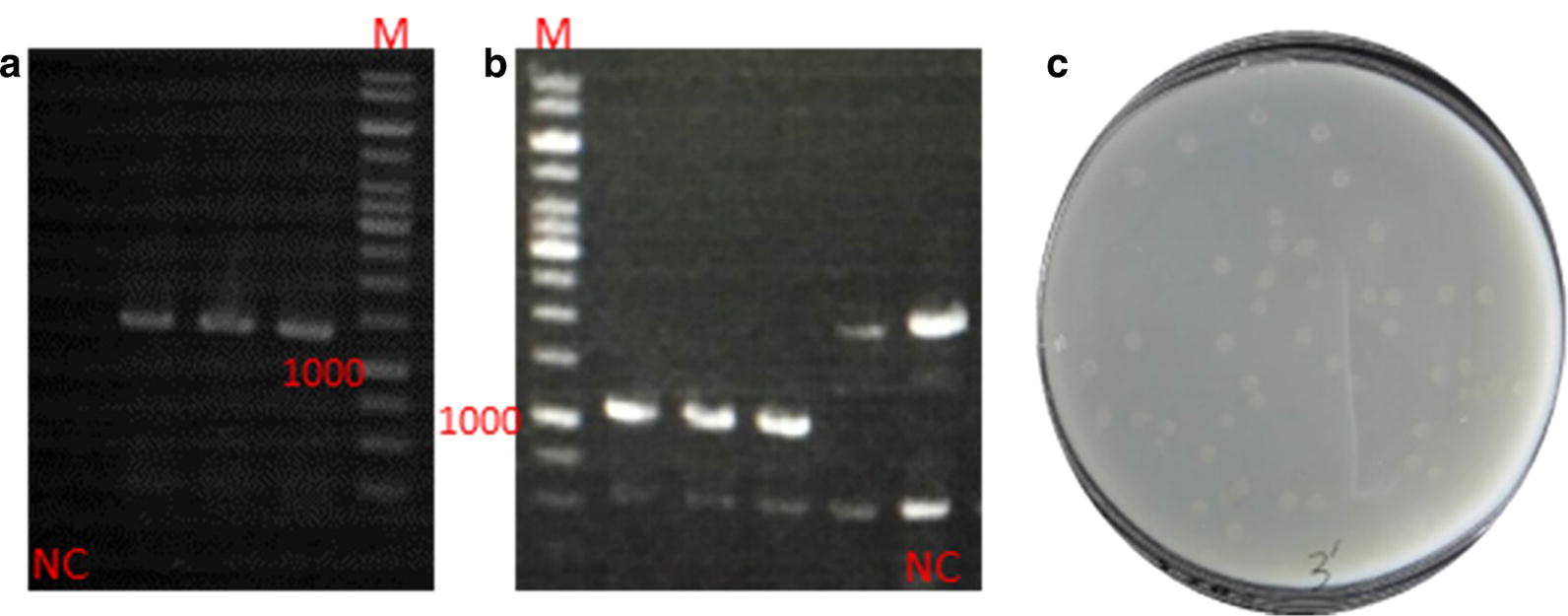
Confirmation of the *aprE* disruption and assaying alkaline protease activity of the mutant. **a** Verification of the single-crossover recombinant with a band of 1250 bp; **b** Verification of the double-crossover mutant with a band of 1100 bp; **c** Alkaline protease activity assay in Buttermilk plate of the *aprE* mutant

In order to better understand the effects of different expression patterns on the production of alkaline protease, the *aprE* gene had to be deleted. As shown in Fig. 4a, the double-crossover mutant BL ΔA, with a 1100 bp band in colony PCR using Apr-VF/Apr-VR, was successfully produced from the right single-crossover strains with a 1250 bp band (colony PCR by Apr-VF/T-R) (Fig. 4b). The enzyme activity of alkaline protease was dramatically reduced (80 U/mL) and there was no transparent zone in the buttermilk plates comprising 4 g/L casein in LB (Fig. 4c). The backcrossing experiment was performed to prove that the observed phenotypes were due to the introduced mutations, and the results confirmed our expectations. Finally, we used the derivative strain BL ΔESA obtained by singly disrupting the three genes, and BL ΔES obtained by singly disrupting the *eps* cluster and *lchAC* mentioned above as the initial strains for further expression optimization.

### Expression optimization of *aprE* in the modified host BL ΔESA

#### Plasmids-mediated expression of *aprE*

**Fig. 5:**
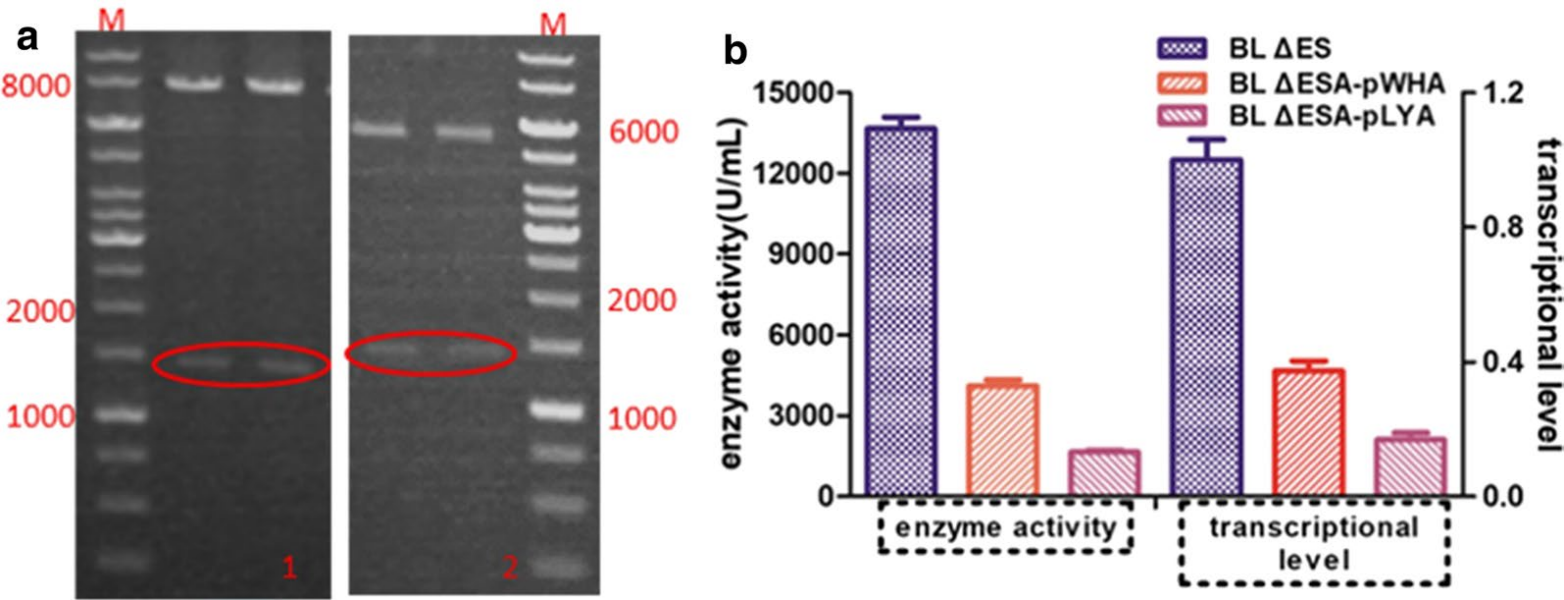
Optimization of plasmid-mediated expression and analysis effect analysis of different strains. **a** The confirmation of the expression vectors by agarose gel electrophoresis. **a**-1, confirmation of pWHA by digestion of *Bam*H I/*Sph* I with about 1500 bp and 8000 bp, **a**-2, Confirmation of pLYA by digestion of *Sac* I/*Kpn* I with about 1500 bp and 6000 bp; **b** Investigation of the *aprE* expression level of different strains. The left Y axis indicated AprE enzyme activity and the right Y axis indicated *aprE* transcriptional level

In order to improve the expression of alkaline protease based on plasmids that are present in multiple copies within the cell and the high compatibility between expression elements and the optimized host, we cloned the endogenous *aprE* expression cassette into the plasmids pWH1520 and pLY-3 resulting in the recombinant plasmids pWHA and pLYA, respectively. The recombinant plasmids were verified by restriction digestion as shown in Fig. 5a-1 and a-2, respectively and were introduced into the BL ΔESA strain via electrotransformation to form the two recombinant strains BL ΔESA-pWHA and BL ΔESA-pLYA, meanwhile the control strains BL ΔESA-pWH1520 and BL ΔESA-pLY-3 were constructed to eliminate the influence of plasmid on cell metabolism maintenance. Unfortunately, the expression of *aprE* in the two recombinant strains was not enhanced through increasing the gene copy number as expected. On the contrary, the alkaline protease was severely affected, which was reflected in both the transcriptional level and enzyme activity (Fig. 5b). As shown in the figure, the AprE enzyme activity of the recombinant strains was about 4106 U/mL (BL ΔESA-pWHA) and 1645 U/mL (BL ΔESA-pLYA), which was much lower than that of the modified host BL ΔES (13,652 U/mL). Transcriptional analysis showed the same trend as the enzyme activity, and the transcriptional level of *aprE* in BL ΔESA-pWHA and BL ΔESA-pLYA was just 0.373 and 0.169 that of the level in BL ΔES, respectively. According to the literature, the backbone of pWH1520 can be stably replicated in *Bacillus* cells (Radha and Gunasekaran 2008), and pLY-3 appears to be more stable a with higher copy number (data not shown). However, according to the results of this study, the transcriptional level of *aprE* decreased with the increasing number of copies of the expression plasmid, which indicated that the endogenous *aprE* cassette preferred to genomic expression. Thus, it was worth a try to further improve AprE production via chromosomal integration of the *aprE* gene.

### Expression of *aprE* in different genomic loci

**Fig. 6:**
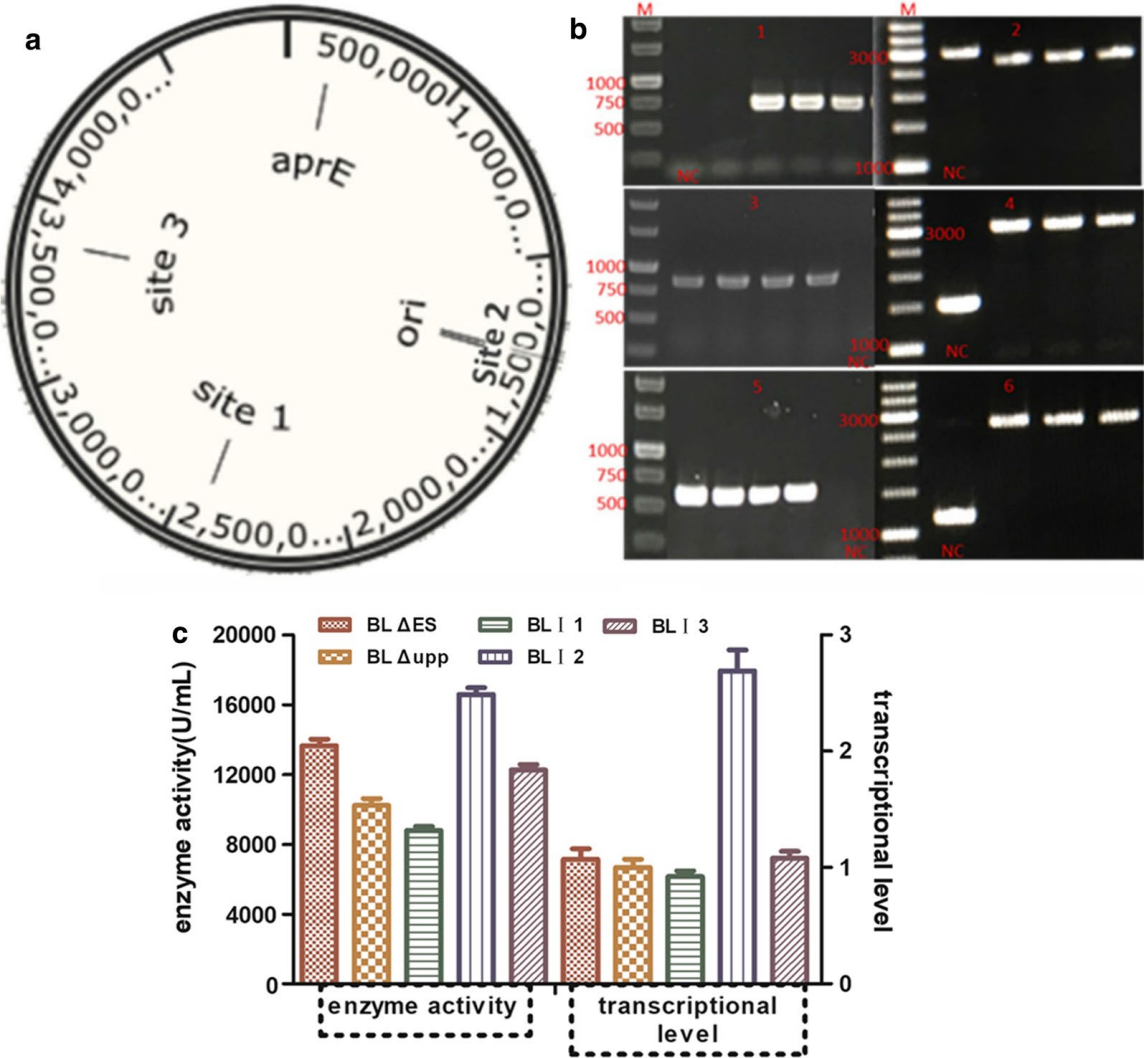
Optimization of *aprE* expression in different genome loci and analysis effect analysis of different strains. **a** Genome locations to be inserted the *aprE* expression cassette in *B. licheniformis* (GenBank Number: CP033218), I-the symmetrical position of the *aprE* (2,419,710-2,422,710 bp), II-near the origin of replication (321,526-322,944 bp), III-the symmetrical position of the replication origin (3,396,863-3,397,860 bp); **b** Confirmation of the integrated mutants by screening the single-crossover recombinant and the double-crossover mutant. BL I1, BL I2, BL I3 correct single-crossover recombinant individually with a band of 700 bp (**b**-1), 900 bp (**b**-3) and 700 bp (**b**-5) (the band can’t be amplified if no precise single-crossover) and correct double-crossover mutant with 2760 bp (**b**-2), 3070 bp (**b**-4) and 2740 bp (**b**-6) band (the band were 3100 bp, 1500 bp and 1200 bp if no correct double-crossover mutant); **c** Investigation of the *aprE* expression level of different integrating strains. The left Y axis indicated AprE enzyme activityand the right Y axis indicated *aprE* transcriptional level

Three specific loci in the genome were selected as shown in Fig. 6a, encompassing locus I in the symmetrical position of the *aprE* by replacing the pullulanase gene (~ 1938 bp), II near the origin of replication and III in the symmetrical position of the replication origin via direct insertion. The three integrative vectors pTUAI1, pTUAI2 and pTUAI3 were individually introduced into BL ΔESA. The mutants were obtained using the gene editing method in this study, and the related verification results are presented in Fig. 6b. In order to verify the single-crossover and double-crossover mutants easily, we simultaneously used the primer pairs Apr-VF/A-R and A-F/Apr-VR because the band of less-than 1000 bp was easier to amplify (A-F/A-R were two oligonucleotides near the two ends of the expression cassette). As can be seen, the single-crossover was confirmed by colony PCR of the recombinant strain using the primer pair Apr-VF1/A-R (oligonucleotides on the right of the left homologous arm in the integration vector) producing a PCR band of about 700-bp band (Fig. 6b-1) and the double-crossover mutant with a 2760-bp band (Fig. 6b-2) were screened, and named *B. licheniformis* I1 (BL I1). While, the *B. licheniformis* I2 (BL I2) and *B. licheniformis* I3 (BL I3) were individually screened by right single-crossover (BL I2, 800 bp and BL I3, 700 bp) and correct double-crossover (BL I2, 3070 bp and BL I3, 2740 bp) as shown in Fig. 6b-3 to b-6.

As indicated in the Fig. 6c, the enzyme activity of chromosomally expressed *aprE* in BL I1, BL I2 and BL I3 was 8784 ± 237 U/mL (lower than that of the optimized strain BL ΔES), 16,504 ± 575 U/mL (greatly higher than that of the optimized strain BL ΔES) and 13,259 ± 359 U/mL (equivalent to that of the optimized strain BL ΔES). BL I2 had a much higher enzyme activity than BL ΔES due to its possible higher dosage of *aprE* gene, as expected. It is particularly noteworthy that the transcriptional level of BL I2 was remarkably improved 1.67-fold compared with the parent strain BL Δupp due to the suitable gene dosage in a chromosomal region adjacent to the origin of replication. Ultimately, a potential industrial workhorse was engineered via genetic modification to remove undesirable wild-type features and expression optimization.

## Discussions

Nowadays, more and more small research teams are attempting to deeply investigate their own favorite strains for special functions, which is often very cumbersome due to a lack of advanced genome editing technologies compared with the well-characterized model microorganisms [29]. Genomic modifications including gene deletion, insertion, and replacement [30], is imperative for the development of *Bacillus* and other Gram-positive bacteria. Although we have demonstrated that the recently developed ultramodern CRISPR/Cas9 system can be established in our host strain [23], there is a problem of complicated construction and verification of knockout vectors, as well as high cost. The markerless gene editing system with the counter-selectable *upp* gene based on a temperature-sensitive plasmid played an important role in genetic modification in the study.

The ultimate goal of the development of gene editing systems is to establish engineering strategies or methods to improve the cellular performance of microbial cell factories. While wild-type bacteria often have interesting and even industrially or medically relevant properties, it is usually difficult to replicate the complex phenotypes of the wild ancestors in the so-called model strains [31]. Therefore, advanced engineering projects often must modify undomesticated strains for the synthesis of desired products. Like other *Bacillus* spp., *B. licheniformis* has many undesirable wild-type properties, such as production of a large amount of foam during fermentation, sporulation under nutrient depletion conditions, and even production of high maintenance metabolism to increase the cells ability of surviving in harsh and competitive environments, which increases the requirements and difficulties in industrial operations [20, 32]. Genetic engineering of undomesticated bacteria can be an effective way to rapidly move the wild bacteria to industrial or model-organism status. We once obtained a *sigF* mutant playing important role in energy conservation, simpler operations and target product controlling effect because of the good industrial properties, such as facilitating the sterilization process, a prolonged stable phase of enzyme production and slower decreasing trend [2]. *B. licheniformis* used in the study can also produce a viscous substance which was identified as EPS secreted in the form of biofilm or capsules to the extracellular medium [28]. EPS synthesis is an integrated result of the cooperative actions of a great many gene products [33]. When the *eps* cluster responsible for the synthesis of EPS was deleted in this study, the viscosity was decreased and non-particulate cell material formed during the fermentation, which might spontaneously increase the dissolved oxygen in the fermentation broth of the EPS-deficient host. The effective accumulation of biomass in turn led to an improvement of alkaline protease synthesis. *B. licheniformis* produces surfactin, an amphiphilic molecule containing a peptide moiety and a β-hydroxy fatty acid side chain [34], whose accumulation at gas–liquid interfaces leads to the formation of foam [34, 35]. The biosynthesis of surfactin is mainly controlled by a non-ribosomal peptide synthase enzyme, SrfAC [36], which catalyzes the conversion of a linear lipoheptapeptide to the cyclic form and controls the release of surfactin [35]. The host used in this study produces a large amount of foam during fermentation, which has serious effects on process control and may lead to contamination. Compared with the parent strain, the mutant BL ΔS produced much less foam that was more sensitive to antifoam, which indicates that lichenysin might be a major mediator of foam formation, and should be studied further in order to thoroughly inhibit foam production.

The regulation of gene expression is another crucial aspect of synthetic biology [37, 38], and effective gene expression is essential for the progression of new host strains toward industrial applications. Sometimes, it is desirable to produce recombinant proteins by using plasmids as genetic carriers because they make it possible to express more mRNA than chromosomally integrated cassettes. In order to improve the *aprE* expression level, we first considered cloning the *aprE* expression cassette into the multicopy plasmids pWH1520 and pLY-3, which are stably replicated in *Bacillus* cells [39]. However, the protease expression of BL ΔESA-pWHA and BL ΔESA-pLYA, respectively harboring pWHA and pLYA, was much lower than that of the optimized strain BL ΔES with a chromosomally integrated cassette. Both the enzyme activity and transcriptional levels of genes expressed from plasmids were relatively low, and the capability of producing alkaline protease decreased along with the increase of plasmid copy number (pWH1520 has a relatively low copy number, pLY-3 has a higher copy number). The transcription was badly limited when the *aprE* was expressed from a plasmid, which may be explained by transcriptional control at the promoter level that may intervene in gene expression [40]. The pathway to transcript formation involves numerous steps, and all of them may be subject to regulation, which is closely related to the multi-subunit structure of DNA-dependent RNA polymerase [41], sigma factors [42], small ligands [43], transcription factors [44], and so on. Transcription requires the precise interaction of every element with an appropriate balance between them [40]. This may explain the observation that when the template DNA concentration substantially increased in plasmids, the transcriptional parts were unable to cooperate with great effectiveness. Thus, the template DNA dosage should be maintained in the proper range and there is more work to be done to keep the identify the correct balance of different factors.

Therefore, we investigated the effect of chromosomal integration in different genomic locations with the correct gene orientation to improve the production of AprE. The copy number of integrated genes can fluctuate due to the special DNA replication mechanism of bacteria, which starts at a fixed position on the genome (origin of replication) [45]. Like other *Bacillus* spp., the orientation of genes on the genome might be correlated with the direction of DNA replication by affecting the transcriptional orientation [46, 47]. In this study, the transcriptional level of *aprE* was strongly increased when the expression cassette was inserted in proximity to the origin of replication. The *aprE* gene dosage may rise at these loci, although heterologous genetic material was stably maintained, because rapidly growing microorganisms can initiate a new round of replication before the previous round has been completed [48]. We also found that as long as the orientation of the *aprE* gene was co-directional with the native genes in the insertion location, the expression was not influenced. The enzyme activity of the engineered host in the study was not consistent with the transcriptional level, so more efforts are needed to improve the translation and secretion levels. Thus, chromosomal expression in *Bacillus* has important advantages for the development of a stable food-grade expression system free of any use of antibiotics during industrial fermentation.

## Conclusions

The host was successfully modified by eliminating the undesirable production of EPS and foam during fermentation found in the wild-type strain *B. licheniformis*. We also successfully performed expression optimization of *aprE* by using its native expression cassette in different genomic loci and plasmids, after which the transcriptional levels and enzyme activity were both remarkably enhanced via chromosomal integration at a location near the origin of replication. We hope that this optimized strategy will enhance *B. licheniformis* as a potential host for efficient protein production.

## Supplementary information

**Additional file 1: Table S1.** Main oligonucleotides used in this study. **Figure S1.** The gene editing procedure based on the temperature-sensitive plasmid. a: construction of the knockout plasmid based on the temperature-sensitive backbone pKSVT by singly inserting the upp cassette and homologous repair arm; b: the whole procedure of the gene editing system from plasmid construction in EC135 to DNA methylation in EC135 pM.Bam and screening of single-crossover recombinant and double-crossover mutants; c: scheme of the markerless gene editing method combining the counter-selectable marker (*upp* gene). A and B represent homologous templates flanking the gene fragment to be deleted. The primer pairs V-F/T-R or T-F/VR was used to verify the single-crossover recombinants after being cultured at 45 °C for 10 h with Kana. The desired mutants were obtained by cultivating the right single-crossover recombinant without antibiotics at 37 °C. 5-FU can facilitate screening the cells undergone the intramolecular recombination, and desired deletion was confirmed by diagnostic PCR using V-F/V-R and DNA sequencing.

## Data Availability

All data generated or analyzed during this study are included in this published article.

## References

[1] Van Dijl JM, Hecker M (2013). *Bacillus subtilis* from soil bacterium to super secreting cell factory. Microb Cell Fact.

[2] Zhou CX, Zhou HY, Zhang HT, Lu FP (2019). Optimization of alkaline protease production by rational deletion of sporulation related genes in *Bacillus licheniformis*. Microb Cell Fact.

[3] Dragos A, Kiesewalter H, Martin M, Hsu CY, Hartmann R, Wechsler T, Eriksen C, Brix S, Drescher K, Stanley-Wall N (2018). Division of labor during biofilm matrix production. Curr Biol.

[4] Voigt B, Schroeter R, Schweder T, Jurgen B, Albrecht D, van Dijl JM, Maurer KH, Hecker M (2014). A proteomic view of cell physiology of the industrial workhorse *Bacillus licheniformis*. J Biotechnol.

[5] Yi GB, Liu Q, Lin JZ, Wang WD, Huang H, Li S (2017). Repeated batch fermentation for surfactin production with immobilized *Bacillus subtilis* BS-37: two-stage pH control and foam fractionation. J Chem Technol Biot.

[6] Fleming AB, Tangney M, Jorgensen PL, Diderichsen B, Priest FG (1995). Extracellular enzyme synthesis in a sporulation-deficient strain of *Bacillus licheniformis*. Appl Environ Microb.

[7] Coutte F, Leclere V, Bechet M, Guez JS, Lecouturier D, Chollet-Imbert M, Dhulster P, Jacques P (2010). Effect of pps disruption and constitutive expression of *srfA* on surfactin productivity, spreading and antagonistic properties of *Bacillus subtilis* 168 derivatives. J Appl Microbiol.

[8] Zhang K, Duan X, Wu J (2016). Multigene disruption in undomesticated *Bacillus subtilis* ATCC 6051a using the CRISPR/Cas9 system. Sci Rep.

[9] Jayamanohar J, Devi PB, Kavitake D, Rajendran S, Priyadarisini VB, Shetty PH (2018). Characterization of alpha-d-glucan produced by a probiont *Enterococcus hirae* KX577639 from feces of south Indian Irula tribals. Int J Biol Macromol.

[10] Liu D, Mao Z, Guo J, Wei L, Ma H, Tang Y, Chen T, Wang Z, Zhao X (2018). Construction, model-based analysis, and characterization of a promoter library for fine-tuned gene expression in *Bacillus subtilis*. ACS Synth Biol.

[11] Liu X, Wang H, Wang B, Pan L (2018). High-level extracellular protein expression in *Bacillus subtilis* by optimizing strong promoters based on the transcriptome of *Bacillus subtilis* and *Bacillus megaterium*. Protein Expr Purif.

[12] Zhou S, Du G, Kang Z, Li J, Chen J, Li H, Zhou J (2017). The application of powerful promoters to enhance gene expression in industrial microorganisms. World J Microbiol Biotechnol.

[13] Mutalik VK, Guimaraes JC, Cambray G, Lam C, Christoffersen MJ, Mai QA, Tran AB, Paull M, Keasling JD, Arkin AP, Endy D (2013). Precise and reliable gene expression via standard transcription and translation initiation elements. Nat Methods.

[14] Sauer C, Ver Loren van Themaat E, Boender LGM, Groothuis D, Cruz R, Hamoen LW, Harwood CR, van Rij T (2018). Exploring the nonconserved sequence space of synthetic expression modules in *Bacillus subtilis*. ACS Synth Biol.

[15] Liao Y, Huang L, Wang B, Zhou F, Pan L (2015). The global transcriptional landscape of *Bacillus amyloliquefaciens* XH7 and high-throughput screening of strong promoters based on RNA-seq data. Gene.

[16] Yang S, Du G, Chen J, Kang Z (2017). Characterization and application of endogenous phase-dependent promoters in *Bacillus subtilis*. Appl Microbiol Biotechnol.

[17] Zhang W, Yang Y, Liu X, Liu C, Bai Z (2019). Development of a secretory expression system with high compatibility between expression elements and an optimized host for endoxylanase production in *Corynebacterium glutamicum*. Microb Cell Fact.

[18] Jeong DE, So Y, Park SY, Park SH, Choi SK (2018). Random knock-in expression system for high yield production of heterologous protein in *Bacillus subtilis*. J Biotechnol.

[19] Browning DF, Busby SJ (2016). Local and global regulation of transcription initiation in bacteria. Nat Rev Microbiol.

[20] Gu Y, Xu X, Wu Y, Niu T, Liu Y, Li J, Du G, Liu L (2018). Advances and prospects of *Bacillus subtilis* cellular factories: from rational design to industrial applications. Metab Eng.

[21] Sauer C, Syvertsson S, Bohorquez LC, Cruz R, Harwood CR, van Rij T, Hamoen LW (2016). Effect of genome position on heterologous gene expression in *Bacillus subtilis*: an unbiased analysis. ACS Synth Biol.

[22] Zhang G, Wang W, Deng A, Sun Z, Zhang Y, Liang Y, Che Y, Wen T (2012). A mimicking-of-DNA-methylation-patterns pipeline for overcoming the restriction barrier of bacteria. PLoS Genet.

[23] Zhou C, Liu H, Yuan F, Chai H, Wang H, Liu F, Li Y, Zhang H, Lu F (2019). Development and application of a CRISPR/Cas9 system for *Bacillus licheniformis* genome editing. Int J Biol Macromol.

[24] Cai D, Chen Y, He P, Wang S, Mo F, Li X, Wang Q, Nomura CT, Wen Z, Ma X, Chen S (2018). Enhanced production of poly-gamma-glutamic acid by improving ATP supply in metabolically engineered *Bacillus licheniformis*. Biotechnol Bioeng.

[25] Prasanna PH, Bell A, Grandison AS, Charalampopoulos D (2012). Emulsifying, rheological and physicochemical properties of exopolysaccharide produced by *Bifidobacterium longum* subsp. infantis CCUG 52486 and *Bifidobacterium infantis* NCIMB 702205. Carbohydr Polym.

[26] Zhang W, Gao W, Feng J, Zhang C, He Y, Cao M, Li Q, Sun Y, Yang C, Song C, Wang S (2014). A markerless gene replacement method for *B. amyloliquefaciens* LL3 and its use in genome reduction and improvement of poly-gamma-glutamic acid production. Appl Microbiol Biotechnol.

[27] Zhu W, Wang Y, Yan F, Song R, Li Z, Li Y, Song B (2018). Physical and chemical properties, percutaneous absorption-promoting effects of exopolysaccharide produced by *Bacillus atrophaeus* WYZ strain. Carbohydr Polym.

[28] Ruas-Madiedo P, De Los Reyes-Gavilán CG (2005). Invited review: methods for the screening, isolation, and characterization of exopolysaccharides produced by lactic acid bacteria. J Dairy Sci.

[29] Liu H, Deutschbauer AM (2018). Rapidly moving new bacteria to model-organism status. Curr Opin Biotechnol.

[30] Dong HN, Zhang DW (2014). Current development in genetic engineering strategies of *Bacillus* species. Microb Cell Fact.

[31] Adams BL (2016). The next generation of synthetic biology chassis: moving synthetic biology from the laboratory to the field. ACS Synth Biol.

[32] Brophy JAN, Triassi AJ, Adams BL, Renberg RL, Stratis-Cullum DN, Grossman AD, Voigt CA (2018). Engineered integrative and conjugative elements for efficient and inducible DNA transfer to undomesticated bacteria. Nat Microbiol.

[33] Barcelos MCS, Vespermann KAC, Pelissari FM, Molina G (2019). Current status of biotechnological production and applications of microbial exopolysaccharides. Crit Rev Food Sci Nutr.

[34] Willenbacher J, Rau JT, Rogalla J, Syldatk C, Hausmann R (2015). Foam-free production of Surfactin via anaerobic fermentation of *Bacillus subtilis* DSM 10(T). AMB Express.

[35] Vater J, Wilde C, Kell H (2009). In situ detection of the intermediates in the biosynthesis of surfactin, a lipoheptapeptide from *Bacillus subtilis* OKB 105, by whole-cell cell matrix-assisted laser desorption/ionization time-of-flight mass spectrometry in combination with mutant analysis. Rapid Commun Mass Spectrom.

[36] Ullrich C, Kluge B, Palacz Z, Vater J (1991). Cell-free biosynthesis of surfactin, a cyclic lipopeptide produced by *Bacillus subtilis*. Biochemistry.

[37] Leavitt JM, Alper HS (2015). Advances and current limitations in transcript-level control of gene expression. Curr Opin Biotechnol.

[38] Lee SY, Kim HU (2015). Systems strategies for developing industrial microbial strains. Nat Biotechnol.

[39] Radha S, Gunasekaran P (2008). Sustained expression of keratinase gene under PxylA and PamyL promoters in the recombinant *Bacillus megaterium* MS941. Bioresour Technol.

[40] Browning DF, Busby SJ (2004). The regulation of bacterial transcription initiation. Nat Rev Microbiol.

[41] Ebright RH (2000). RNA polymerase: structural similarities between bacterial RNA polymerase and eukaryotic RNA polymerase II. J Mol Biol.

[42] Maeda H, Fujita N, Ishisama A (2000). Competition among seven *Escherichia coli* sigma subunits: relative binding affinities to the core RNA polymerase. Nucleic Acids Res.

[43] Chatterji D, Ojha AK (2001). Revisiting the stringent response, ppGpp and starvation signaling. Curr Opin in Microbiol.

[44] Martınez-Antonio A, Collado-Vides J (2003). Identifying global regulators in transcriptional regulatory networks in bacteria. Curr Opin in Microbiol.

[45] Couturier E, Rocha EP (2006). Replication-associated gene dosage effects shape the genomes of fast-growing bacteria but only for transcription and translation genes. Mol Microbiol.

[46] Tillier ER, Collins RA (2000). The contributions of replication orientation, gene direction, and signal sequences to base-composition asymmetries in bacterial genomes. J Mol Evol.

[47] Zeigler DR, Donald DH (1990). Orientation of genes in the *Bacillus subtilis* chromosome. Genetics.

[48] Sousa C, de Lorenzo V, Cebolla A (1997). Modulation of gene expression through chromosomal positioning in *Escherichia coli*. Microbiology.

