# Supplementary material for: Optimized expression and enhanced production of alkaline protease by genetically modified Bacillus licheniformis 2709
Source: Microb Cell Fact. 2020 Feb 24;19:45. doi: 10.1186/s12934-020-01307-2 (PMC7041084; doi:10.1186/s12934-020-01307-2)
Supplement: Supplementary file 1 — Additional file 1: Table S1. Main oligonucleotides used in this study. Figure S1. The gene editing procedure based on the temperature-sensitive plasmid. a: construction of the knockout plasmid based on the temperature-sensitive backbone pKSVT by singly inserting the upp cassette and homologous repair arm; b: the whole procedure of the gene editing system from plasmid construction in EC135 to DNA methylation in EC135 pM.Bam and screening of single-crossover recombinant and double-crossover mutants; c: scheme of the markerless gene editing method combining the counter-selectable marker (upp gene). A and B represent homologous templates flanking the gene fragment to be deleted. The primer pairs V-F/T-R or T-F/VR was used to verify the single-crossover recombinants after being cultured at 45 °C for 10 h with Kana. The desired mutants were obtained by cultivating the right single-crossover recombinant without antibiotics at 37 °C. 5-FU can facilitate screening the cells undergone the intramolecular recombination, and desired deletion was confirmed by diagnostic PCR using V-F/V-R and DNA sequencing. [file 12934_2020_1307_MOESM1_ESM.docx]

Table S1 Main oligonucleotides used in this study

| Primer name | Sequence (5'–3') | Purpose |
| --- | --- | --- |
| Apr-LF | GAATTCCTGCAGCCCGGGGGATCCGCAAGCACCATCAATACGCC | *Bam*HI*/Sac*II  PCR of homologous template for deletion of *aprE* gene |
| Apr-LR | AGCTTCCCAAAGCAGAAGCGGAATCGCTG |  |
| Apr-RF | CGCTTCTGCTTTGGGAAGCTCCTTCTACTATGG |  |
| Apr-RR | CTTTTCTACGAGCTCCACCGCGGCATAGGACTTGGCGGGTATCTG |  |
| Eps-LF | GAATTCCTGCAGCCCGGGGGATCCGGCCTCGTCAGGATATTCCTG | *Bam*HI*/Sac*II  PCR of homologous template for deletion of *eps cluster* |
| Eps-LR | CGACGTTTCCGGCGCTGAAAGTCGAATTAAAG |  |
| Eps-RF | TTTCAGCGCCGGAAACGTCGGGGATCTATTG |  |
| Eps-RR | CTTTTCTACGAGCTCCACCGCGGCCGCCTGTCATACGTGAACAG |  |
| Lch-LF | GAATTCCTGCAGCCCGGGGGATCCTTCCTGTGTCAGCATGCAAAC | *Bam*HI*/Sac*II  PCR of homologous template for deletion of *srfAC* gene |
| Lch-LR | CGTTGAGCACGATCTGAATTTGCTTGCTTCACC |  |
| Lch-RF | AATTCAGATCGTGCTCAACGTGGTATGATGC |  |
| Lch-RR | CTTTTCTACGAGCTCCACCGCGGTGAAACGTCCGGTTCAAGTC |  |
| IL-F1 | GAATTCCTGCAGCCCGGGGGATCCCTTCAGAGCATGGAAATCATTGC | *Bam*HI*/Sac*II  PCR of homologous template and *aprE* expression cassette for integration into site 1 of genome |
| IL-R1 | GGTGAAAGATAAACGTACGCTTGTCATCCCC |  |
| IR-F1 | AGCTTTTTCTGTTTTGAAGAAGATGCGGACACT |  |
| IR-R1 | CTTTTCTACGAGCTCCACCGCGGCATAGGTCCCGATGCCGTTC |  |
| ap-F1 | GCGTACGTTTATCTTTCACCCGTTTCTGTATGC |  |
| ap-R1 | TCTTCAAAACAGAAAAAGCTAGTGCTAAAAACACTAGC |  |
| IL-F2 | GAATTCCTGCAGCCCGGGGGATCCCCAATACCGCGCTTACCCTATC | *Bam*HI*/Sac*II  PCR of homologous template and *aprE* expression cassette for integration into site 2 of genome |
| IL-R2 | GGTGAAAGATAGGGGTGAAATGCCAATCGA |  |
| IR-F2 | AGCTTTTTCTACCCACACCTCATCCCCGC |  |
| IR-R2 | CTTTTCTACGAGCTCCACCGCGGCTGTAAGCAAGCTTTGATCCGGAG |  |
| ap-F2 | TTTCACCCCTATCTTTCACCCGTTTCTGTATGC |  |
| ap-R2 | AGGTGTGGGTAGAAAAAGCTAGTGCTAAAAACACTAGC |  |
| IL-F3 | GAATTCCTGCAGCCCGGGGGATCCGATGCTTCCACCGGAAGTTG | *Bam*HI*/Sac*II  PCR of homologous template and *aprE* expression cassette for integration into site 3 of genome |
| IL-R3 | GGTGAAAGATCATCTTGTCTTAAATCCATTCACAAAA |  |
| IR-F3 | AGCTTTTTCTAAGGCGCCTTAATTGGCG |  |
| IR-R3 | CTTTTCTACGAGCTCCACCGCGGGACAAGATGGCGTTACTTGCG |  |
| ap-F3 | AGACAAGATGATCTTTCACCCGTTTCTGTATGC |  |
| ap-R3 | AAGGCGCCTTAGAAAAAGCTAGTGCTAAAAACACTAGC |  |
| Apr-VF | GTACTGTGCAGCGCTTCCC | diagnostic PCR of the mutation and sequence---*aprE* |
| Apr-VR | GAAATGGGGCACTATGTGATGA |  |
| Ep -VF | CTGGAAGACGATCCGATCGTG | diagnostic PCR of the mutation and sequence---*eps cluster* |
| Eps-VR | CGGATTCAGCATTTGGATGAAG |  |
| Lch-VF | CGTCGATCAGGATCGTATGCT | diagnostic PCR of the mutation and sequence---*srfAC* |
| Lch-VR | ATGAATGTCATCATCGGCAGGT |  |
| I-VF1 | GAGAATGGAATCATGAGCGATTG | diagnostic PCR of the mutation and sequence-integration 1 |
| I-VR1 | GACGCCAAACTTCGTCAAGC |  |
| I-VF2 | GATTTGCCTATTTCCCAGCCTAAC | diagnostic PCR of the mutation and sequence- integration 2 |
| I-VR2 | GACGGGACGAACACCGATATG |  |
| I-VF3 | GAGGGCGATGAACTCGTTTATG | diagnostic PCR of the mutation and sequence- integration 3 |
| I-VR3 | CCGTTTCATTAAAAGCACCTCAC |  |
| T-F | CCGACTGCGCAAAAGACATAAT | diagnostic PCR of single-crossover recombinant |
| T-R | CGTAATCTGCTGCTTGCAAACA |  |
| A-F | GGTCTGATCAATGTCGAAGCTGC | diagnostic PCR of single-crossover by combining with I-VR and I-VF, respectively |
| A-R | TGCGGCCTTGTCGATCATC |  |
| 16S-F | CGTTGCTCCGTCAGACTTTC | fluorogenic quantitative PCR of 16s rRNA |
| 16S-R | GGGCTAATACCGGATGCTTG |  |
| AP-F | CGGATCTTCAGGAAACACG | fluorogenic quantitative PCR of *apr*E |
| AP-R | ATGAGGAGAAGCCATTGACG |  |

**Note: The underlined bases are sites for restriction digest**


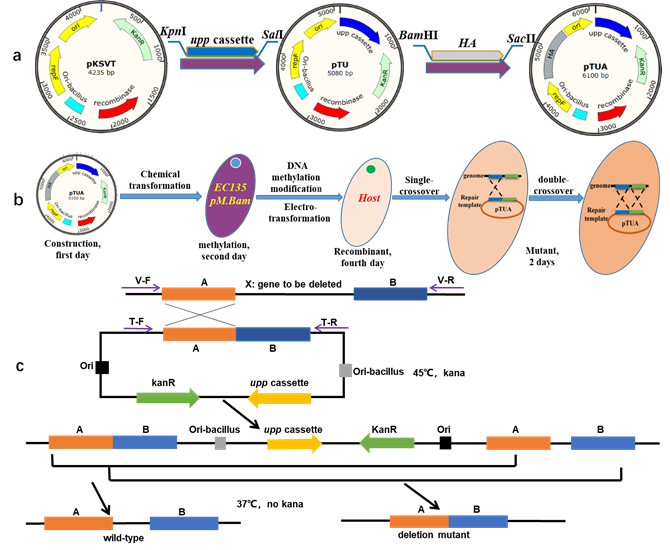


**Fig. S1 The gene editing procedure based on the temperature-sensitive plasmid.**

a: Construction of the knockout plasmid based on the temperature-sensitive backbone pKSVT by singly inserting the upp cassette and homologous repair arm;

b: The whole procedure of the gene editing system from plasmid construction in EC135 to DNA methylation in EC135 pM.Bam and screening of single-crossover recombinant and double-crossover mutants;

c: Scheme of the markerless gene editing method combining the counter-selectable marker (*upp* gene). A and B represent homologous templates flanking the gene fragment to be deleted. The primer pairs V-F/T-R or T-F/VR was used to verify the single-crossover recombinants after being cultured at 45 ℃ for 10 h with Kana. The desired mutants were obtained by cultivating the right single-crossover recombinant without antibiotics at 37 ℃. 5-FU can facilitate screening the cells undergone the intramolecular recombination, and desired deletion was confirmed by diagnostic PCR using V-F/V-R and DNA sequencing.
